# Supplementary material for: DMRforPairs: identifying Differentially Methylated Regions between unique samples using array based methylation profiles
Source: BMC Bioinformatics. 2014 May 15;15:141. doi: 10.1186/1471-2105-15-141 (PMC4046028; doi:10.1186/1471-2105-15-141)
Supplement: Additional file 2 — DMRforPairs output for the comparison of A431-MCF7 and NA17018-NA17105. Please start from the HTML files in each folder. Available via the BMC Bioinformatics website. [file 1471-2105-15-141-S2.zip › 1394847754114233_MOESM2_ESM/A431_MCF7/figures/34463.pdf]

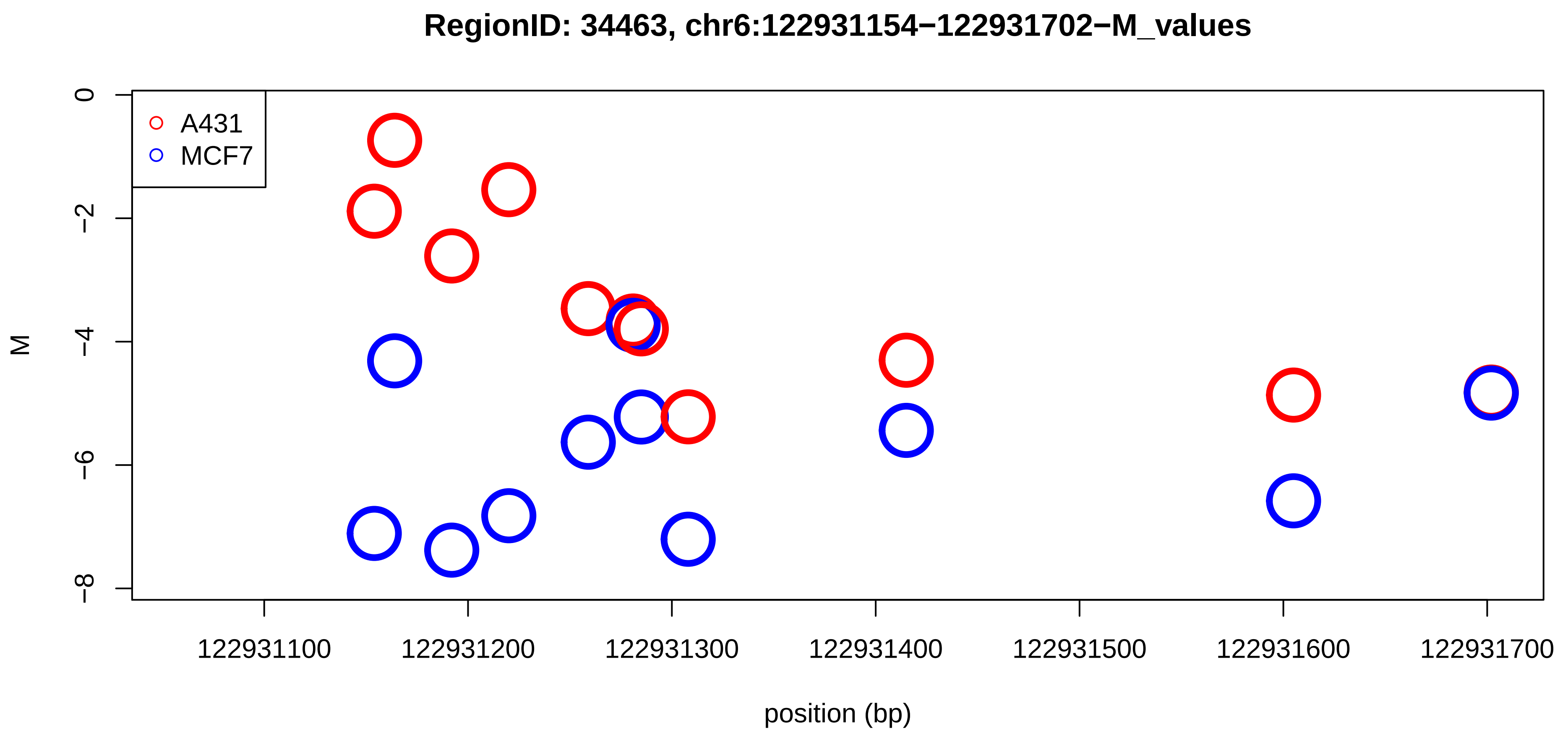

RegionID: 34463, chr6:122931154−122931702−M_values
A431
MCF7
M
0
−2
−4
−6
−8
122931100
122931200
122931300
122931400
122931500
122931600
122931700
position (bp)

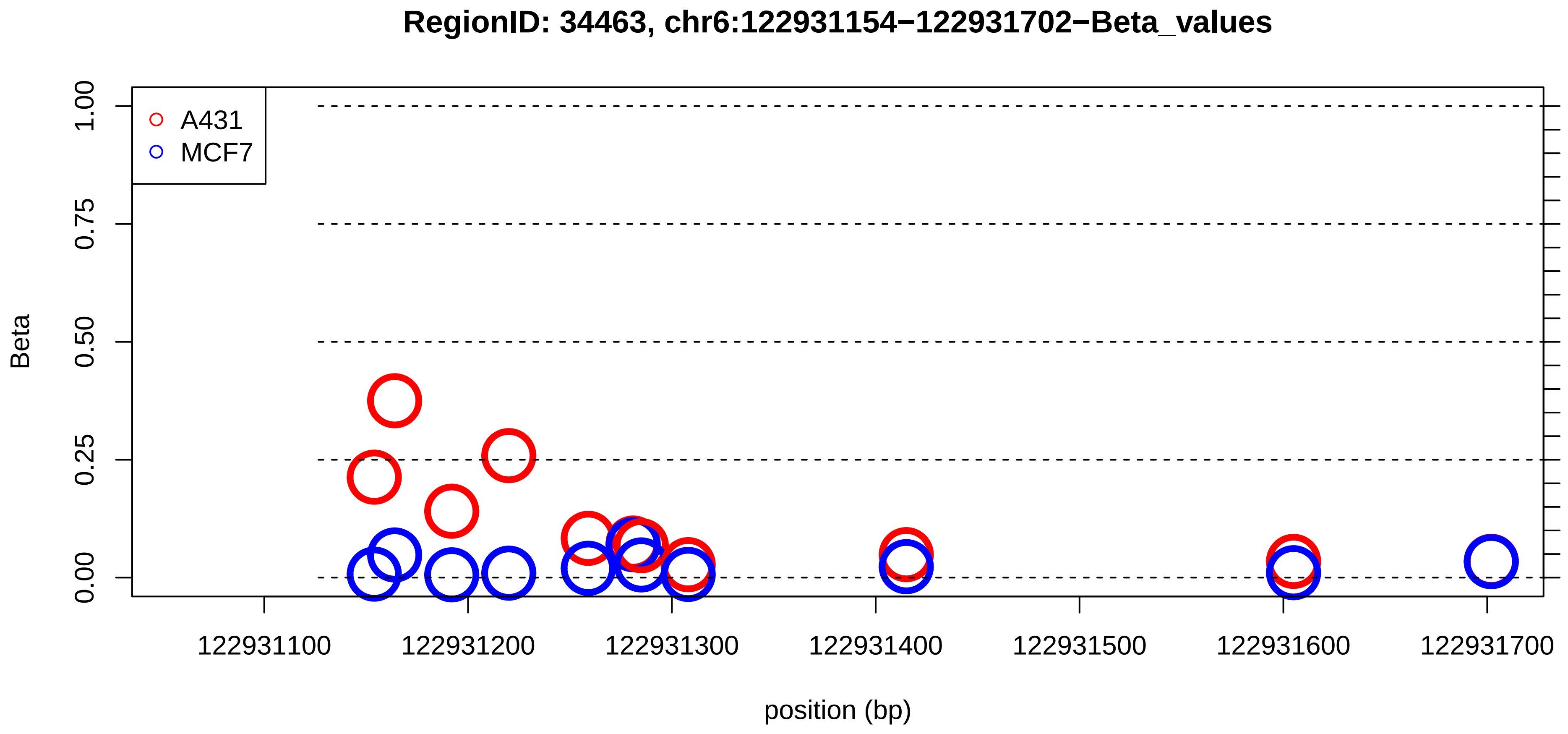

RegionID: 34463, chr6:122931154−122931702−Beta_values
A431
MCF7
Beta
1.00
0.75
0.50
0.25
0.00
122931100
122931200
122931300
122931400
122931500
122931600
122931700
position (bp)

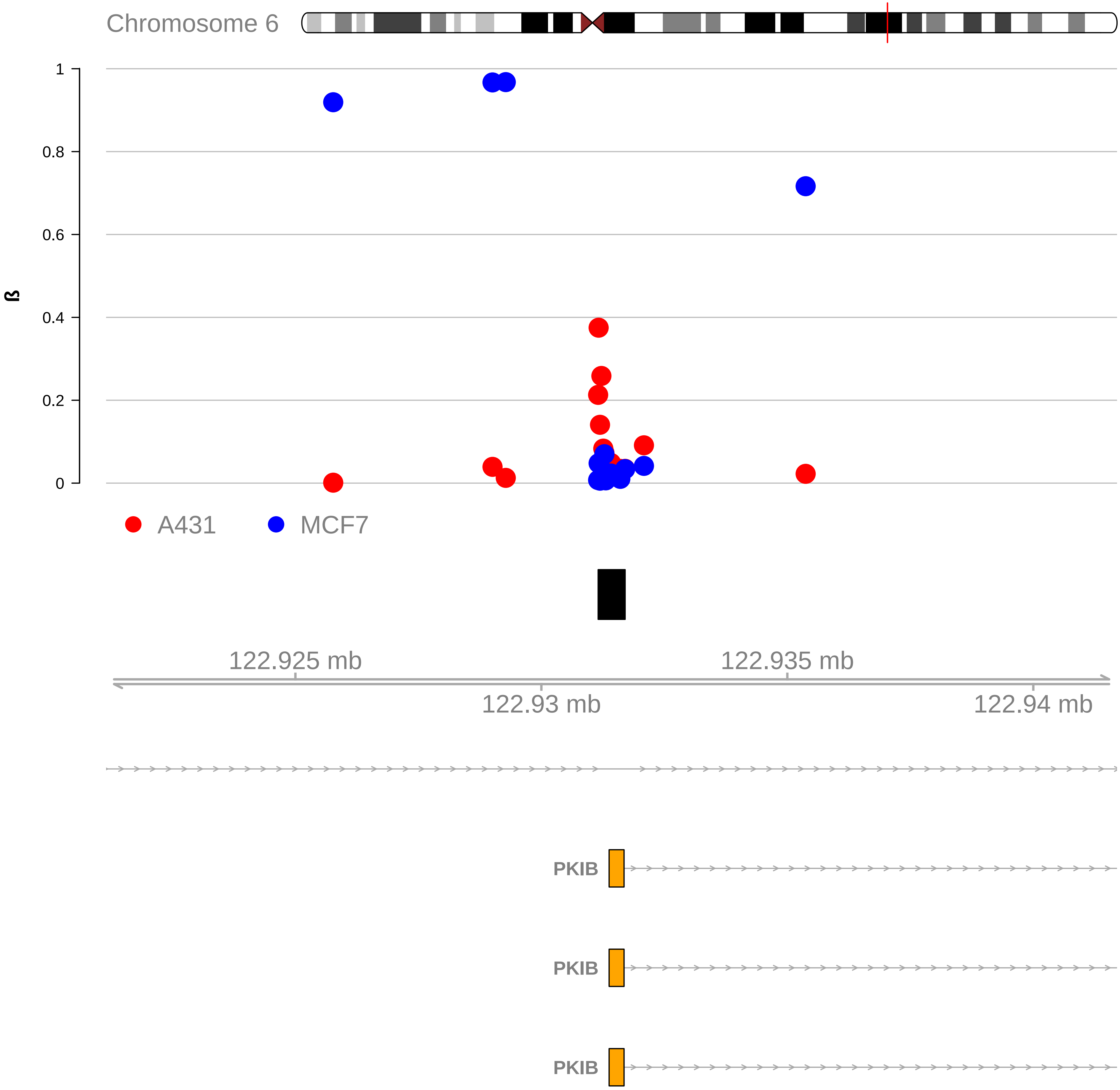

Chromosome 6
1
0.8
0.6
0.4
0.2
0
ß
A431
MCF7
122.925 mb
122.935 mb
122.93 mb
122.94 mb
PKIB
PKIB
PKIB
